# Stochasticity in space, persistence in time: genetic heterogeneity in harbour populations of the introduced ascidian *Styela plicata*

**DOI:** 10.7717/peerj.2158

**Published:** 2016-06-23

**Authors:** Mari-Carmen Pineda, Beatriz Lorente, Susanna López-Legentil, Creu Palacín, Xavier Turon

**Affiliations:** 1 Department of Animal Biology and Biodiversity Research Institute (IRBIO), University of Barcelona, Barcelona, Spain; 2 Sustainable Coastal Ecosystems & Industry in Tropical Australia, Australian Institute of Marine Science, Townsville, Queensland, Australia; 3 Department of Biology & Marine Biology and Center for Marine Science, University of North Carolina Wilmington, Wilmington, North Carolina, United States; 4 Department of Marine Ecology, Centre for Advanced Studies of Blanes (CEAB-CSIC), Blanes, Girona, Spain

**Keywords:** *COI*, Harbours, Ascidians, Spatio-temporal genetic structure, Introduced species

## Abstract

Spatio-temporal changes in genetic structure among populations provide crucial information on the dynamics of secondary spread for introduced marine species. However, temporal components have rarely been taken into consideration when studying the population genetics of non-indigenous species. This study analysed the genetic structure of *Styela plicata,* a solitary ascidian introduced in harbours and marinas of tropical and temperate waters, across spatial and temporal scales. A fragment of the mitochondrial gene Cytochrome Oxidase subunit I (*COI*) was sequenced from 395 individuals collected at 9 harbours along the NW Mediterranean coast and adjacent Atlantic waters (> 1,200 km range) at two time points 5 years apart (2009 and 2014). The levels of gene diversity were relatively low for all 9 locations in both years. Analyses of genetic differentiation and distribution of molecular variance revealed strong genetic structure, with significant differences among many populations, but no significant differences among years. A weak and marginally significant correlation between geographic distance and gene differentiation was found. Our results revealed spatial structure and temporal genetic homogeneity in *S. plicata*, suggesting a limited role of recurrent, vessel-mediated transport of organisms among small to medium-size harbours. Our study area is representative of many highly urbanized coasts with dense harbours. In these environments, the episodic chance arrival of colonisers appears to determine the genetic structure of harbour populations and the genetic composition of these early colonising individuals persists in the respective harbours, at least over moderate time frames (five years) that encompass ca. 20 generations of *S. plicata*.

## Introduction

The increase in maritime traffic and aquaculture activities in recent decades has fostered the spread of non-indigenous marine species (NIS) across the globe (Carlton, 1996; Galil, 2000; Grosholz, 2002; Zenetos et al., 2012). At the same time, the field of invasion genetics has become a well-established discipline (Holland, 2000; Geller, Darling & Carlton, 2010; Darling, 2015) and genetic tools have proved invaluable for understanding crucial aspects of the invasion process, including cryptic diversity, introduction pathways, and connectivity among native and introduced populations (Rius & Darling, 2014; Viard & Comtet, 2015; Rius et al., 2015).

International traffic among large commercial ports is a common pathway for the spread of NIS into new and often distant areas (i.e., pre-border dispersal, sensu Forrest, Gardner & Taylor, 2009). Further expansion to nearby areas is facilitated by smaller vessels mostly used for fishing and recreational activities (Wasson et al., 2001; Darbyson et al., 2009; Goldstien, Schiel & Gemmell, 2010; Davidson et al., 2010). Therefore, small harbours and marinas play an important role in the spread of NIS at the local level (secondary spread or post-border dispersal, Forrest, Gardner & Taylor, 2009). In highly urbanized coastal areas, dense networks of harbours and artificial structures can act as stepping-stone strongholds for the propagation of introduced species (Glasby et al., 2007; Dafforn, Glasby & Johnston, 2012; López-Legentil et al., 2015; Airoldi et al., 2015). These networks provide unique opportunities for the study of dispersal mechanisms and processes occurring during secondary spread of NIS.

The Mediterranean is the largest enclosed sea on Earth and supports intense, international maritime traffic (Kaluza et al., 2010; Keller et al., 2011). Consequently, the Mediterranean Sea has been invaded by many NIS (Streftaris, Zenetos & Papathanassiou, 2005; Streftaris & Zenetos, 2006; Galil, 2009; Coll et al., 2010) and shipping is among the leading vectors of introductions (Zenetos et al., 2012; Galil et al., 2014). In addition, Mediterranean coasts are highly urbanized and support a dense network of harbours and artificial structures, in particular the NW region (Airoldi & Beck, 2007). Therefore, the NW Mediterranean represents an ideal system to study the role of harbours in post-border processes of NIS dispersal (López-Legentil et al., 2015; Airoldi et al., 2015).

An unexpected conclusion of many marine invasion genetic studies is that founder effects are not always the norm, contrary to theoretical considerations. Instead, introduced populations often display similar or even higher levels of genetic diversity than native populations (reviewed in Rius et al., 2015), with important implications for their success (Tsutsui et al., 2000; Barrett & Schluter, 2008). Recurrent introductions from different locations and/or introductions of large numbers of individuals often explain the high genetic diversity of introduced populations (Frankham, 2005; Roman & Darling, 2007). In particular, recurrent introductions are expected in primary entry points such as ports with international traffic. However, populations in small harbours and marinas are likely subject to high stochasticity in the arrival of individuals, resulting in the low levels of genetic diversity previously reported (Dupont et al., 2007a; Pérez-Portela, Turon & Bishop, 2012; Rius & Shenkar, 2012). In such cases, the combined effects of bottlenecks, genetic drift, and adaptation to novel environments (Sakai et al., 2001; Strayer et al., 2006; Keller & Taylor, 2008) may yield important temporal changes in the genetic composition of these populations.

Despite the importance of temporal dynamics for understanding introduction processes in the sea (Rius et al., 2015), most studies to date only address spatial differentiation in the genetic structure of NIS. Among the few studies that have investigated temporal variation, some report marked decreases in genetic diversity over time (Pérez-Portela, Turon & Bishop, 2012), while others documented short-term changes in allele frequencies, within a context of sustained high genetic diversity (Paz et al., 2003; Guardiola, Frotscher & Uriz, 2012; Reem et al., 2013; Karahan et al., 2016; Pineda et al., 2016). To our knowledge, only Dupont, Bernas & Viard (2007b) reported temporal genetic homogeneity across age groups of an introduced gastropod. Goldstien et al. (2013) demonstrated how temporal sampling can provide a better understanding of introduction dynamics and improve the identification of sources of introduced populations.

Ascidians have prominent examples of marine introduced species, which often thrive in artificial habitats (Lambert & Lambert, 2003; Lambert, 2007; López-Legentil et al., 2015; Ordóñez et al., 2015; Ordóñez et al., 2016) causing economic losses (Aldred & Clare, 2014). The limited natural dispersal capabilities of ascidians (Svane & Young, 1989; David, Marshall & Riginos, 2010) makes them dependent on artificial transport for long-distance dispersal and an exemplary model for the study of introduction processes (Zhan et al., 2015). *Styela plicata* is a solitary ascidian that has been translocated around the globe for centuries (Pineda, López-Legentil & Turon, 2011), which has blurred any signal about its native area (presumably the NW Pacific, de Barros, da Rocha & Pie, 2009; Pineda, López-Legentil & Turon, 2011). The type-specimen was collected from a ship hull in Philadelphia (NE USA) and its present distribution encompasses warm-temperate areas of the Atlantic and Indo-Pacific oceans, from approximately 45°N to 38°S (de Barros, da Rocha & Pie, 2009). *S. plicata* can withstand drastic changes in temperature and salinity (Thiyagarajan & Qian, 2003; Pineda, Turon & López-Legentil, 2012) and tolerates high levels of pollutants in the water (Galletly, Blows & Marshall, 2007; Pineda, Turon & López-Legentil, 2012). Not surprisingly, *S. plicata* is a conspicuous member of the fouling communities in harbours and artificial structures throughout the world.

In this study, *S. plicata* was used as a model to test the spatio-temporal dynamics of populations inhabiting small to medium-size harbours and marinas across > 1,200 km of Mediterranean coast (Iberian Peninsula) and adjacent Atlantic waters. Contrary to other introduced ascidians in this area (e.g., *Microcosmus squamiger*, Ordóñez et al., 2013), *S. plicata* does not occur outside of ports and confined environments, thus its dispersal among localities relies on human transport. We sampled the same populations at two time points separated by five years. Considering that *S. plicata* grows to maturity in three months and features a continuous reproductive cycle in the study area (Tursi & Matarrese, 1981; Pineda, López-Legentil & Turon, 2013), this temporal scale encompassed at least 20 generations. The investigated harbours have mostly local traffic and are likely seeded by occasional interchange of NIS among neighbouring harbours, as well as interchange with more distant, larger ports that act as entry points. Thus, we hypothesized that the high stochasticity of these seeding events and the low number of individuals that can be transported by small vessels at a given time will result in a patchwork-like distribution of genetic variability, which will in turn be highly dynamic in time as a result of bottlenecks, drift, and further occasional interchanges. Unravelling the genetic signatures of these processes will provide insight into the secondary spread of NIS in the area and contribute to the broader knowledge of introduced species dynamics in highly urbanized coasts.

## Material and Methods

### Sampling

**Figure 1:**
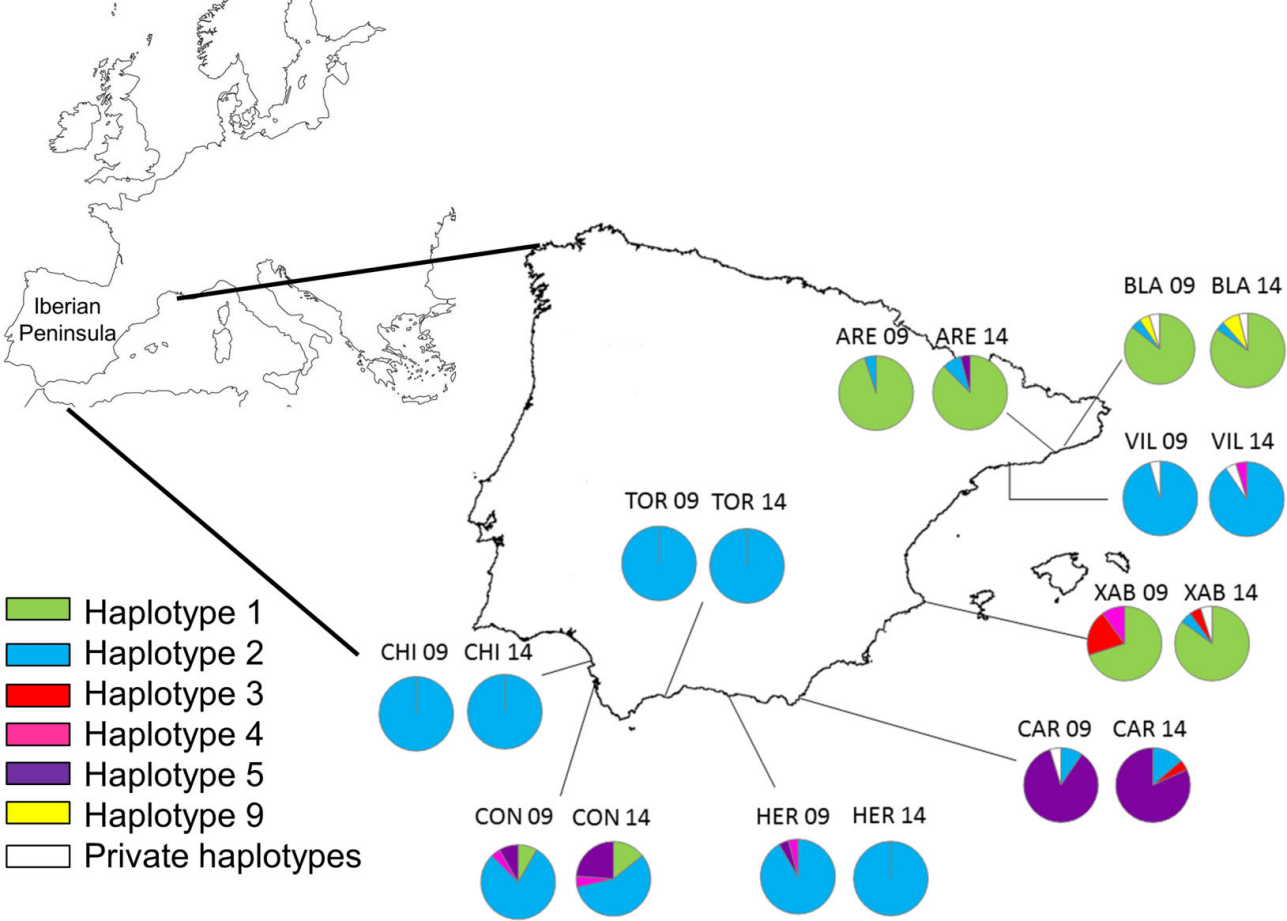
Map of the Iberian Peninsula (NW Mediterranean) showing the sampling sites of *Styela plicata*. Pie charts represent haplotype frequencies for the *COI* gene in each population analysed in 2009 and 2014. Private haplotypes are shown in white. Population codes as in Table 1.

Nine localities along the Spanish coast (Iberian Peninsula) were sampled: seven on the Mediterranean side and two on the Atlantic shores, near the Strait of Gibraltar (Fig. 1). These localities consisted of small to medium-size harbours, mostly with only short-range fishing fleets and/or recreational vessels (Table 1). The harbours were sampled in 2009 and 2014 by collecting specimens attached to ropes and buoys (at least 5 m apart from each other), at 0–2 m depth. The ascidians were dissected immediately upon collection, and tissue close to the buccal siphon was preserved in absolute ethanol. Samples were kept at −20 °C until processed.

**Table 1:** Locality name. code. geographical region and GPS position for the populations of *Styela plicata* analysed in this study. Harbour size is based on the surface of enclosed waters within the perimeter of the harbour (m^2^) calculated with the polygon tool on Google Earth Pro (Google Inc.).

Locality	Code	Region	Harbour size (×10^3^ m^2^)	Coordinates
Blanes	BLA	Mediterranean	108	41°40′27″N; 02°47′45″E
Arenys de Mar	ARE	Mediterranean	160	41°34′35″N; 02°33′31″E
Vilanova i la Geltrú	VIL	Mediterranean	360	41°12′53″N; 01°44′10″E
Xàbia	XAB	Mediterranean	91	38°47′52″N; 00°11′06″E
Carboneras	CAR	Mediterranean	53	36°59′22″N; 01°53′56″W
La Herradura	HER	Mediterranean	23	36°43′38″N; 03°43′37″W
Torre Ladrones	TOR	Mediterranean	16	36°29′08″N; 04°44′28″W
Conil	CON	Atlantic	54	36°17′42″N; 06°08′17″W
Chipiona	CHI	Atlantic	87	36°44′45″N; 06°25′46″W

### DNA extraction and sequencing

DNA was extracted from muscle or branchial tissue with REDExtract-N-AmpTissue PCR Kit (Sigma-Aldrich, St. Louis, MO, USA). A fragment of the mitochondrial Cytochrome Oxidase I (*COI*) gene was amplified using the universal primers LCO1490 and HCO2198 (Folmer et al., 1994). Amplifications were performed in a final volume of 20 μL using 10 μL of REDExtract-N-amp PCR reaction mix (Sigma-Aldrich, St. Louis, MO, USA), 0.8 μL of each primer (10 μM), and 2 μL of template DNA. The PCR program consisted of an initial denaturing step at 94 °C for 2 min, 30 amplification cycles (denaturing at 94 °C for 45 s, annealing at 50 °C for 45 s and extension at 72 °C for 50 s), and a final extension at 72 °C for 6 min, on a PCR System 9700 (Applied Biosystems). PCR products were purified using MultiScreen® filter plates (Millipore), labelled using BigDye® Terminator v.3.1 (Applied Biosystems) and sequenced on an ABI 3730 Genetic Analyser (Applied Biosystems) at the Scientific and Technological Centres of the University of Barcelona, Spain (CCiTUB). Other samples were directly sent for purification and sequencing to Macrogen Inc. (Seoul, South Korea). Sequences were edited and aligned using BioEdit® v.7.0.5.3 (Hall, 1999).

### Genetic analyses

Number of alleles (*Nh*), haplotype diversity (*Hd*), and nucleotide diversity (π) were computed with DnaSP v.5 (Librado & Rozas, 2009). Two estimates of allelic differentiation between populations at each sampling year were used: the *F_ST_* estimator of Weir & Cockerham (1984), based on allele frequencies and calculated with Arlequin v 3.0 (Excoffier, Laval & Schneider, 2005); and the adjusted D_est_ estimate described by Jost (2008), and obtained with SPADE (Chao & Shen, 2010). Both estimators varied between 0 (no differentiation) and 1 (complete differentiation). The use of *F_ST_*-like estimators has been criticized on the basis that it is dependent on the variability of the marker used (Jost, 2008; Jakobsson, Edge & Rosenberg, 2013), and it is advisable to use estimators independent of within population diversity, such as D_est_, in combination with traditional *F_ST_*-like statistics (Meirmans & Hedrick, 2011; Verity & Nichols, 2014). The significance of *F_ST_* values was calculated (permutation tests, 10,000 replicates), and a correction for multiple comparisons was applied following the Benjamini-Yekutieli false discovery rate (FDR) (Narum, 2006). For D_est_, the mean and SE values obtained with SPADE from 10,000 bootstrap replicates were used to calculate confidence intervals (using a normal approximation) with a FDR-corrected probability. A value of D_est_ was deemed significant when the confidence interval around its mean did not contain 0.

In order to complement these differentiation methods based on allelic frequencies, we used two approaches that explicitly consider the spatial distribution of alleles across samples. First, we performed a spatial analysis of shared alleles (SAShA, Kelly et al., 2010), which tests the average geographic distance between co-occurrences of alleles against its expectation under panmixia. Based on results from the previous tests (see Results), this analysis was performed pooling the samples for both years. We used the SAShA 1.0 software and performed 10,000 permutations of data for assessing significance. Individual alleles were also analysed separately, which may be useful when common alleles mask a potential non-random distribution of less common alleles. In a second approach, estimates of gene flow (in number of migrants per generation) based on the frequency of private alleles (Slatkin, 1985; Barton & Slatkin, 1986) were performed between the localities studied at both years using the web version of the GENEPOP 4.2 software (Raymond & Rousset, 1995). Again, the analysis of private alleles may uncover patterns masked by abundant and widespread alleles.

For temporal comparisons, differentiation values (*F_ST_* and D_est_) were computed for each population between sampling years. A correlation between these values among pairs of populations at both years was also calculated. Finally, an analysis of the molecular variance (AMOVA) was performed using haplotype frequencies with Arlequin, grouping the populations per sampled year, and its significance was tested by running 10,000 permutations of the data.

Mantel tests were also computed to correlate genetic and geographic distances separately at each year. The shortest distances by sea between points were calculated using Google Earth (Google Inc.). Mantel tests were performed with Arlequin for *F_ST_* and with the R package ade4 (function *mantel-rtest*) (Dray & Dufour, 2007) for D_est_, and their significance was tested by permutation (10,000 replicates).

The dataset was used to construct a median-joining network using Network v.4.5.1.6 (Bandelt, Forster & Röhl, 1999). A maximum likelihood tree was also computed using *Styela gibsii* as an outgroup (GenBank accession number HQ916447) with Mega v6.06 (Tamura et al., 2013), and the significance of the branches was tested by 10,000 bootstrap replicates. The General Time Reversible (GTR) model with proportion of invariable sites (+I) and rate variation among sites (+G) was selected for tree-building using jModelTest v.2.1.7 (Posada, 2008) and the Akaike Information Criterion.

## Results

Twenty to twenty-six individuals were sequenced per locality in 2009 and 2014 (Table 2), resulting in a total of 395 sequences with a final length after alignment and trimming of 580 bp. These sequences corresponded to 12 haplotypes featuring 28 variable positions. Half were private haplotypes (Table 2) from a single locality and time point. Between 1 and 4 haplotypes were found in each population. For consistency with the global dataset of Pineda, López-Legentil & Turon (2011), the same haplotype numbers presented in that study were used for identical sequences. New haplotypes were labelled from 23 onwards, as there were 22 haplotypes described in Pineda, López-Legentil & Turon (2011). Six of our sequences (haplotypes 1, 2, 3, 4, 5 and 9) were already identified by Pineda, López-Legentil & Turon (2011), of which 5 (haplotypes 1, 2, 3, 4 and 5) were also found by Maltagliati et al. (2015) in Italian waters (from both eastern and western shores). Haplotype frequencies per population and year are presented in Supplemental Information (Table S1). All new sequences obtained in this study have been deposited in GenBank (accession numbers KU878146–KU878151).

**Table 2:** Diversity measures for the studied populations of *Styela plicata*. Population codes as in Table 1. Year of sampling. Number of individuals analysed per population (N). Haplotype (Hd) and nucleotide (π) diversity and their corresponding standard deviations in parentheses. Number of haplotypes per population (*Nh*) with private haplotypes in parentheses.

Locality	Year	N	Hd	SD	π	SD	Number of haplotypes
BLA	2009	21	0.271	(±0.124)	0.00049	(±0.00060)	4(1)
	2014	26	0.286	(±0.112)	0.00052	(±0.00062)	4(1)
ARE	2009	20	0.1	(±0.088)	0.00017	(±0.00034)	2
	2014	24	0.235	(±0.109)	0.00269	(±0.00185)	3
VIL	2009	22	0.090	(±0.080)	0.00015	(±0.00032)	2(1)
	2014	21	0.185	(±0.110)	0.00082	(±0.00082)	3(1)
XAB	2009	20	0.484	(±0.112)	0.00417	(±0.00263)	3
	2014	20	0.284	(±0.128)	0.00136	(±0.00115)	4(1)
CAR	2009	21	0.266	(±0.119)	0.00796	(±0.00454)	3(1)
	2014	23	0.383	(±0.119)	0.01075	(±0.00591)	4
HER	2009	24	0.163	(±0.099)	0.00287	(±0.00194)	3
	2014	21	0	–	0	–	1
TOR	2009	23	0	–	0	–	1
	2014	21	0	–	0	–	1
CON	2009	24	0.373	(±0.119)	0.00522	(±0.00314)	4
	2014	20	0.594	(±0.097)	0.01186	(±0.00651)	4
CHI	2009	24	0	–	0	–	1
	2014	20	0	–	0	–	1
Total		395					12

Overall, haplotype and nucleotide diversity were low (0.206 ± 0.042 and 0.0027 ± 0.0009, respectively, mean ± SD) (Table 2). The highest haplotype diversity appeared in Conil (CON) in 2014 (0.594 ± 0.097), while a single haplotype (*Hd* = 0) was found in La Herradura (HER) in 2014 and in Torre Ladrones (TOR) and Chipiona (CHI) at both years. No clear geographic or temporal trend was apparent for haplotype diversity, with some populations showing higher values in 2009 and others in 2014 (Table 2).

Haplotype 2 was the most abundant and widespread (only absent in Xàbia in 2009), followed by haplotype 1, which was dominant in Blanes (BLA), Arenys de Mar (ARE), and Xàbia (XAB). Haplotype 5 was the third most frequent and the dominant haplotype in Carboneras (CAR). The remaining haplotypes were found only in 6 or less individuals (Fig. 1; Table S1). The haplotype frequencies in the two years were quite similar for the three common haplotypes (Fig. S1), while they varied in the less abundant alleles, of which three were found exclusively in 2009 and another three only in 2014 (Fig. S1).

**Figure 2:**
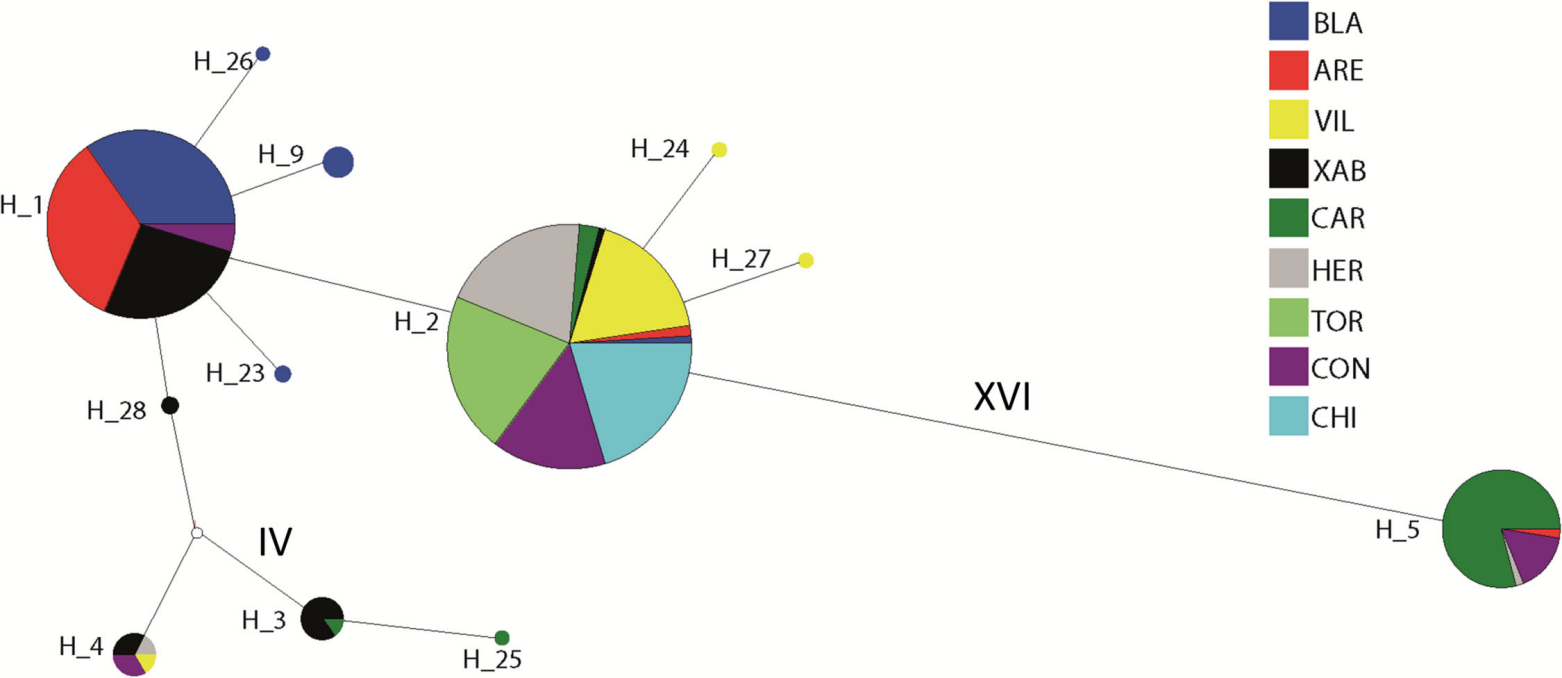
Network of haplotypes. Median-joining haplotype network for *Styela plicata* using *COI* sequences. Area of circles is approximately proportional to the number of individuals found for each haplotype. Partitions inside the circles represent the proportion of each population within each haplotype. Lines between circles represent one mutational step, except where number of steps is indicated with roman numerals.

**Figure 3:**
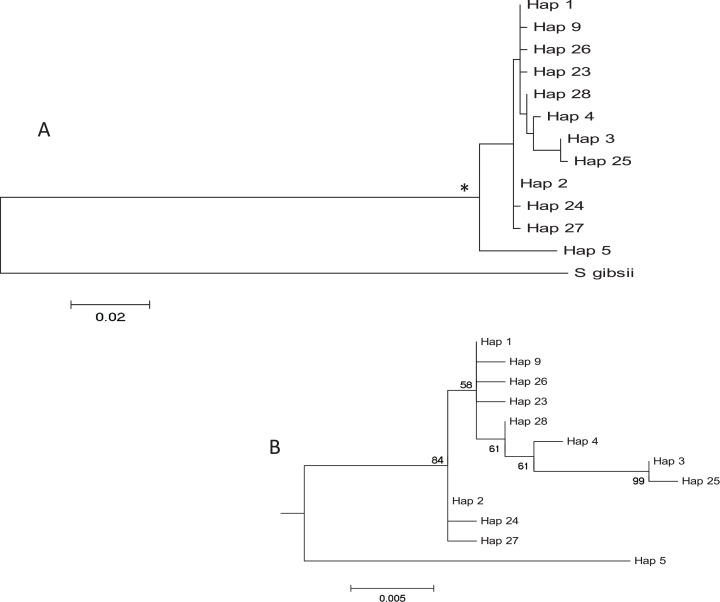
Tree of *COI* haplotypes. (A) Maximum Likelihood tree of partial *COI* sequences based on the GTR model. The congeneric species *Styela gibbsii* was used as an outgroup. (B) Detail of the *Styela plicata* branch (*) in (A) with bootstrap values indicated when > 50%.

The haplotype network revealed that haplotype 5 was most divergent, separated by 16 mutational steps from the remaining haplotypes, which were grouped more closely together, albeit with some divergent sequences (e.g. haplotypes 3 and 25, Fig. 2). Accordingly, the maximum likelihood tree (Fig. 3) showed a highly supported branch comprising all sequences except for haplotype 5, which was set apart in a different branch.

The results of the spatial differentiation analyses using *F_ST_* and D_est_ are shown in Table 3. Mean values for both years were 0.481 ± 0.044 for *F_ST_* and 0.586 ± 0.052 for D_est_ (mean ± SE). A majority of pairwise comparisons were significant (*F_ST_*: 64% in 2009, 72% in 2014; D_est_: 64% in 2009 and in 2014). Overall, Vilanova i la Geltrú (VIL) was the locality that showed fewer significantly different comparisons with other populations, while Carboneras (CAR) showed significant differentiation in all cases. In contrast, the values of differentiation between years for the nine localities were extremely low and non-significant in all cases (*F_ST_* = 0.007 ± 0.004, D_est_ = 0.005 ± 0.003, mean ± SE) (Table 3).

**Figure 4:**
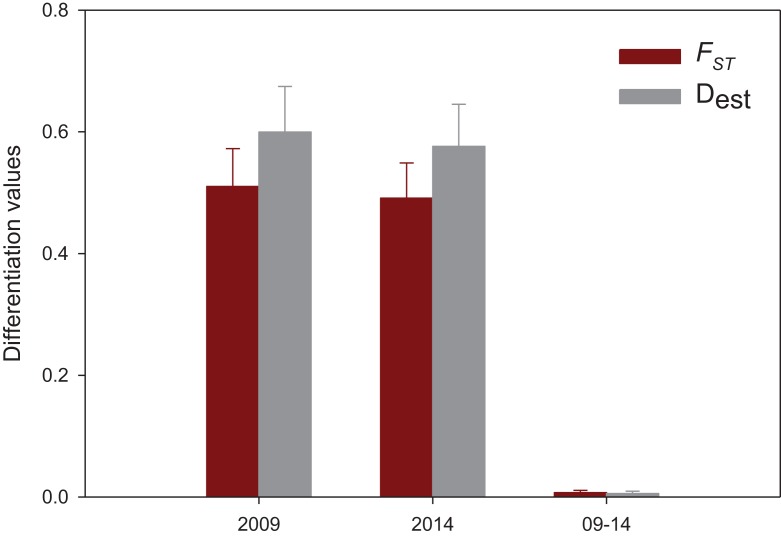
Mean population differentiation. Mean values of population differentiation (*F_ST_* in red; D_est_ in grey) between population pairs in 2009, 2014 and between both years. Error bars correspond to SE.

**Figure 5:**
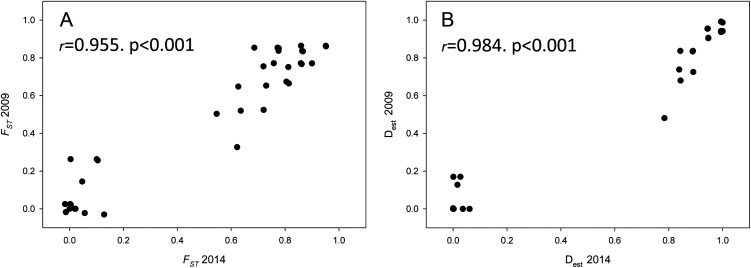
Population differentiation correlations. Correlation of both population differentiation estimators (*F_ST_* (A) and D_est_ (B)) between 2009 and 2014 for all population pairs. Correlation coefficients and associated *p*-values are indicated.

Population differentiation means per year and between years are plotted in Fig. 4, which clearly reflects the gap between differentiation measures among localities and those between years for the same locality. There is also a highly significant correlation between *F_ST_* and D_est_ values for both years (correlation coefficients *r* > 0.95, Fig. 5), indicating that the differentiation level for any given population pair was maintained over the period studied. Figure 5 clearly shows two groups of pairwise comparisons separated by a gap. Some population pairs exhibited low differentiation values, including geographically close populations (for instance, most populations pairs around the Strait of Gibraltar) and also distant populations (for instance, Xàbia (XAB) with the two northernmost populations). A majority of population pairs, however, showed high differentiation values at both years (Fig. 5).

**Table 3:** Genetic differentiation between populations and time-point pairs (2009 vs. 2014) for *COI*. D_est_ values are shown above the diagonal and *F_ST_* values below the diagonal (significant pairwise comparisons after FDR correction in bold and underlined). Some comparisons resulted in slightly negative values, which were set to 0. Population codes as in Table 1.

	BLA09	ARE09	VIL09	XAB09	CAR09	HER09	TOR09	CON09	CHI09	BLA14	ARE14	VIL14	XAB14	CAR14	HER14	TOR14	CON14	CHI14
BLA09		0	**0.944**	0.036	**0.994**	**0.944**	**0.945**	**0.839**	**0.945**	0								
ARE09	0		***0.*947**	0.061	**0.994**	**0.947**	**0.947**	**0.844**	**0.947**		0							
VIL09	**0.812**	**0.900**		**1**	**0.889**	0	0	0.016	0			0						
XAB09	0.056	0.128	**0.720**		**1**	**0.994**	**1**	**0.891**	**1**				0.018					
CAR09	**0.730**	**0.814**	**0.805**	**0.626**		**0.843**	**0.890**	**0.784**	**0.890**					0				
HER09	**0.776**	**0.859**	0	**0.686**	**0.758**		0.002	0.001	0.002						0.002			
TOR09	**0.863**	**0.951**	0.002	**0.772**	**0.859**	0.020		0.027	0							0		
CON09	**0.636**	**0.721**	0.047	**0.546**	**0.622**	0.004	0.101		0.027								0.031	
CHI09	**0.866**	**0.952**	0.004	**0.776**	**0.862**	0.022	0	0.104										0
BLA14	0										0	**0.954**	0	**0.992**	**0.955**	**0.955**	**0.738**	**0.955**
ARE14		0								0		**0.904**	0	**0.937**	**0.906**	**0.906**	**0.680**	**0.906**
VIL14			0							**0.751**	**0.771**		**0.941**	**0.834**	0.003	0.003	0.128	0.003
XAB14				0.027						0	0	**0.755**		**0.987**	**0.942**	**0.942**	**0.725**	**0.942**
CAR14					0					**0.653**	**0.664**	**0.674**	**0.648**		**0.837**	**0.837**	**0.481**	**0.837**
HER14						0.015				**0.837**	**0.864**	0.025	**0.854**	**0.771**		0	0.170	0
TOR14							0			**0.837**	**0.864**	0.025	**0.854**	**0.771**	0		0.170	0
CON14								0.022		**0.520**	**0.524**	0.145	**0.503**	**0.327**	**0.263**	**0.263**		0.170
CHI14									0	**0.834**	**0.861**	0.022	**0.850**	**0.767**	0	0	**0.257**	

**Figure 6:**
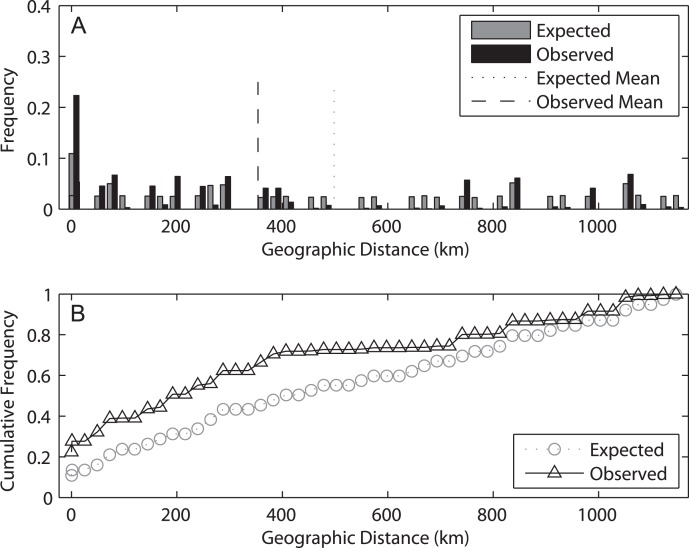
Results of the spatial analysis of shared alleles. The geographic distances observed between co-occurring alleles and those expected under panmixia are given in (A) in the form of histograms and in (B) as cumulative frequency plots. The observed and expected mean distances are indicated in the upper graph.

The spatial analysis of shared alleles (SAShA), combining information of both years, showed a significantly restricted spatial distribution of the alleles in our samples (expected mean distance between co-occurrences: 497.98 km, observed mean: 353.63 km, p < 0.001) (Fig. 6). These results imply that alleles occur more closely together than expected by chance, indicating an overall restriction to gene flow. A more detailed analysis allele per allele (only those distributed among at least two localities can be used in this approach) (Fig. S2) revealed that this pattern is consistent among alleles, with the exception of haplotype 4, which is more evenly distributed among localities. On the other hand, an analysis based on the frequency of private alleles revealed a non-negligible migration rate between localities, and the estimate was lower (1.139 migrants) for 2009 than for 2014 (2.201 migrants). This difference relates to the higher mean frequency of private alleles in 2009 (0.078) than in 2014 (0.053).

The results of the AMOVA grouping populations by sampling year showed that most genetic variability occurred between populations in a given year (74.26%, p < 0.001) and within populations (33.64%, p < 0.001) (Table 4). In contrast, the variance explained by the factor year was negative and not significant. The Mantel tests showed in general marginally significant results (*F_ST_*-2009: *r* = 0.287, p = 0.065; *F_ST_*-2014: *r* = 0.252, p = 0.086; D_est_-2009: *r* = 0.273 p = 0.066; D_est_-2014: *r* = 0.252, p = 0.084), indicating an overall relationship between genetic and geographic distances, albeit with the geographic component explaining little variance in genetic structure.

**Table 4:** Analysis of molecular variance (AMOVA) for the *COI* gene.

Source of variation	*df*	Sum of squares	Variance components	% variation	*p*-value
Between years	1	0.347	−0.02415 Va	−7.90	0.848
Between populations within years	16	81.256	0.22693 Vb	74.26	< 0.001
Within populations	377	38.762	0.10282 Vc	33.64	< 0.001
Total	394	120.365	0.30560		

## Discussion

Our setting is representative of many highly urbanized coasts along the Mediterranean Sea and elsewhere, with a dense network of harbours and marinas of different sizes. We focused specifically on small to medium-size harbours, which mainly host short-range fishing fleets and recreational boats. These are typically sites of secondary dispersal for introduced species (post-border dispersal sensu Forrest, Gardner & Taylor, 2009). In a study of populations of *Styela clava* in New Zealand, Goldstien, Schiel & Gemmell (2010) showed that large ports and marinas can have separate dynamics. Large ports with commercial and overseas cruise activities are interspersed among our sampling areas (Barcelona, Tarragona, Valencia, Cartagena, and Algeciras have the highest volumes of traffic) and can act as initial entry points of introduced species from other seas.

Through the analysis of the genetic structure of populations of an introduced ascidian over ca. 1,200 km of coastline at two time points five years apart, we have found significant spatial differentiation, but negligible temporal variation. This was contrary to our prediction of high variability related to both space and time in this species that relies on human transport for dispersion between harbours. The system was less dynamic than anticipated, showing temporal persistence in the genetic composition of the populations over a period of time that encompassed at least 20 generations of the species. This time frame has also provided ample opportunities for interchange via vessel movements, considering that fishing activities are continuous and that recreational traffic is very active in the studied area, particularly in summertime.

In another population of *Styela plicata*, Pineda et al. (2016) documented a much more dynamic scenario with frequent gains and losses of microsatellite alleles. That population, however, was located in an unstable habitat in the Atlantic Intracoastal Waterway (North Carolina, USA), subject to periodic flooding and die-off episodes, which contrasts with the apparent stability of the harbours analysed here, at least over the temporal scale analysed. Importantly, Pineda et al. (2016) used both microsatellite and *COI* sequence datasets, and the former was more informative than the latter. However, changes in low frequency *COI* haplotypes were detected in Pineda et al. (2016) after die-off episodes, suggesting that mitochondrial sequence information alone is enough to detect substantial temporal changes in genetic structure.

Two major clades of *COI* have been described for *Styela plicata* (Pineda, López-Legentil & Turon, 2011) and most of our sequences belonged to haplogroup 1. The divergent haplotype 5 was the only representative of haplogroup 2, confirming its presence in the Mediterranean Sea (Maltagliati et al., 2015). Overall, some geographically close localities displayed very different haplotype composition (e.g., Vilanova i la Geltrú and Arenys de Mar), while some widely separated populations were genetically similar. Other localities, like Carboneras, exhibited a haplotype composition completely different from all other populations. Therefore, the spatial pattern was complex and heterogeneous, albeit with a tendency towards the dominance of haplotype 1 in the North and haplotype 2 in the South of the studied area. This tendency explains the marginally significant Mantel tests, as North-South comparisons were also the most distant ones.

The dominance of a few haplotypes resulted in populations being relatively similar or highly differentiated, depending on whether they shared one or several of these common haplotypes, with few populations showing intermediate levels of variability (Fig. 5). The analysis of statistics based on spatial distribution of alleles showed more subtle patterns. For instance, the spatial analysis of shared alleles indicated a geographic span of allele co-occurrences smaller than expected, which suggested some degree of stepping stone dispersal among localities (Kelly et al., 2010). The inference based on private alleles, on the other hand, showed a small but non-negligible estimate of migration between localities, and changes in private alleles were also detected between years. Thus, the genetic composition of the populations may in fact change over time, but likely at temporal scales much larger than the one studied here.

The results concerning spatial variability in ascidians inhabiting harbours and artificial substrates showed a diversity of outcomes. Our finding of a significant spatial component is consistent with results obtained in previous studies (e.g., *Styela clava*, Dupont et al., 2009; Dupont et al., 2010; *Botryllus schlosseri*, López-Legentil, Turon & Planes, 2006), while other introduced ascidians did not show spatial differentiation at regional scales (e.g., *Ciona* spp., Zhan, Macisaac & Cristescu, 2010, *Microcosmus squamiger*, Rius, Pascual & Turon, 2008; Ordóñez et al., 2013). In *Styela plicata*, previous studies have shown significant spatial structure between harbour populations (Torkkola, Riginos & Liggins, 2013; Maltagliati et al., 2015), although the degree of differentiation could vary latitudinally as a function of temperature (David, Marshall & Riginos, 2010).

Haplotype richness (1–4 haplotypes) and gene diversity values (mean *Hd* of ca. 0.2) were generally low in the sampled populations and lower than reported for the same marker in other introduced ascidians in harbours (e.g., *Botryllus schlosseri*, López-Legentil, Turon & Planes, 2006; Lejeusne et al., 2010; *Microcosmus squamiger*, Rius, Pascual & Turon, 2008; *Diplosoma listerianum*, Pérez-Portela et al., 2013). In other cases, evidence for bottlenecks and low genetic diversity was found associated with introduction events (e.g., *Corella eumyota*, Dupont et al., 2007a; *Didemnum vexillum*, Stefaniak et al., 2012; *Perophora japonica*, Pérez-Portela, Turon & Bishop, 2012). In further instances, a wide range of genetic diversities has been found among introduced populations of some species (e.g., *Styela clava*, Goldstien et al., 2011). In *Styela plicata*, low values of diversity, similar to those found here, were reported for populations from Australia and New Zealand (Torkkola, Riginos & Liggins, 2013), while in a study of 15 harbours in Italy (including some big ports with international traffic), widely different values of haplotype diversity were found (Maltagliati et al., 2015). The low genetic diversity observed here is consistent with the idea that the studied populations are seeded by small number of individuals, likely associated to local boating activities. However, the lack of significant changes in the genetic structure of all investigated populations over time is unexpected and suggests that interchange with other populations is sparse in time, and that genetic drift alone in inherently small populations does not suffice to modify allele frequencies, at least at the temporal scale surveyed (5 years).

In conclusion, our results confirm the stochastic nature of colonization of small harbours and marinas by introduced species traveling as ship fouling. Recurrent introductions do not seem to be frequent in these harbours, preventing genetic homogenization over space and enabling the persistence of haplotypes in a given location over time, barring major perturbations that result in die-offs (Pineda et al., 2016). Our study area is representative of many highly urbanized coasts with dense harbours. In these environments, the episodic chance arrival of early colonisers appears to determine the structure of the harbour populations and the genetic composition of these early colonising individuals persists in the respective harbours, at least over moderate time frames encompassing tens of generations.

## Supplemental Information

10.7717/peerj.2158/supp-1 Supplemental Information 1 Figure S1. Allele frequencies in the two years studied. Note different y-axis for the abundant (left of break) and the rare haplotypes (right of break). Asterisks indicate haplotypes that were private (appeared in only one locality) at the corresponding year.

10.7717/peerj.2158/supp-2 Supplemental Information 2 Figure S2. Spatial Analysis of Shared Alleles. Frequency distributions of observed (triangles) and expected (circles) distances between co-occurrences of the alleles present in more than one locality, both sampling dates pooled. *p*-values indicate significant differences obtained through permutation of data.

10.7717/peerj.2158/supp-3 Supplemental Information 3 Table S1. Haplotype frequencies observed for the COI gene at each locality and year.

## Additional Information and Declarations

### DNA Deposition

The following information was supplied regarding the deposition of DNA sequences: GenBank accession numbers KU878146–KU878151.

### Data Deposition

The following information was supplied regarding data availability: The raw data has been supplied as Supplemental Dataset Files.
