# Supplementary figures and images for: Stochasticity in space, persistence in time: genetic heterogeneity in harbour populations of the introduced ascidian Styela plicata
Source: PeerJ. 2016 Jun 23;4:e2158. doi: 10.7717/peerj.2158 (PMC4924124; doi:10.7717/peerj.2158)

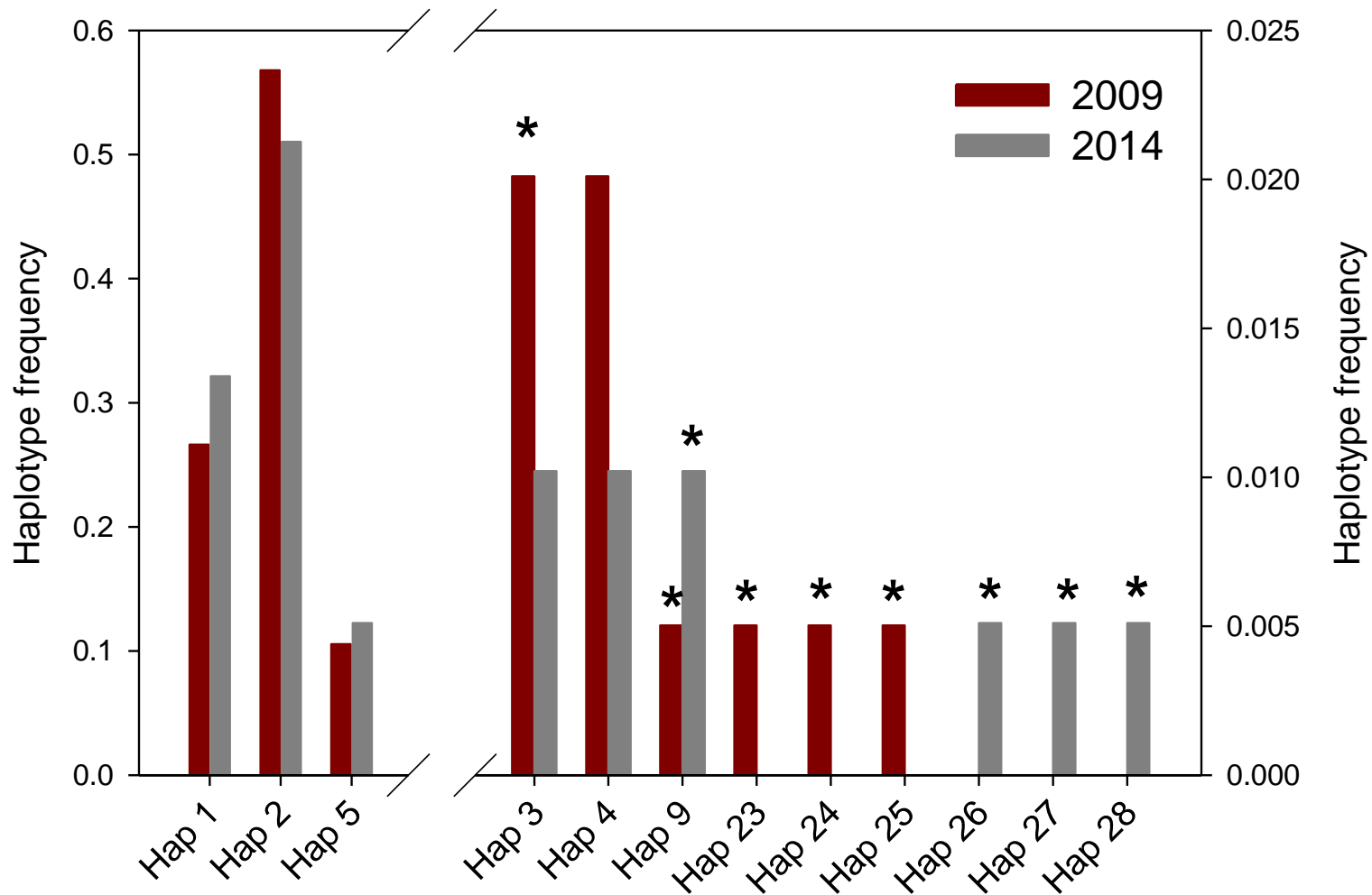

Supplement: Supplemental Information 1 — Note different y-axis for the abundant (left of break) and the rare haplotypes (right of break). Asterisks indicate haplotypes that were private (appeared in only one locality) at the corresponding year. [file peerj-04-2158-s001.pdf]

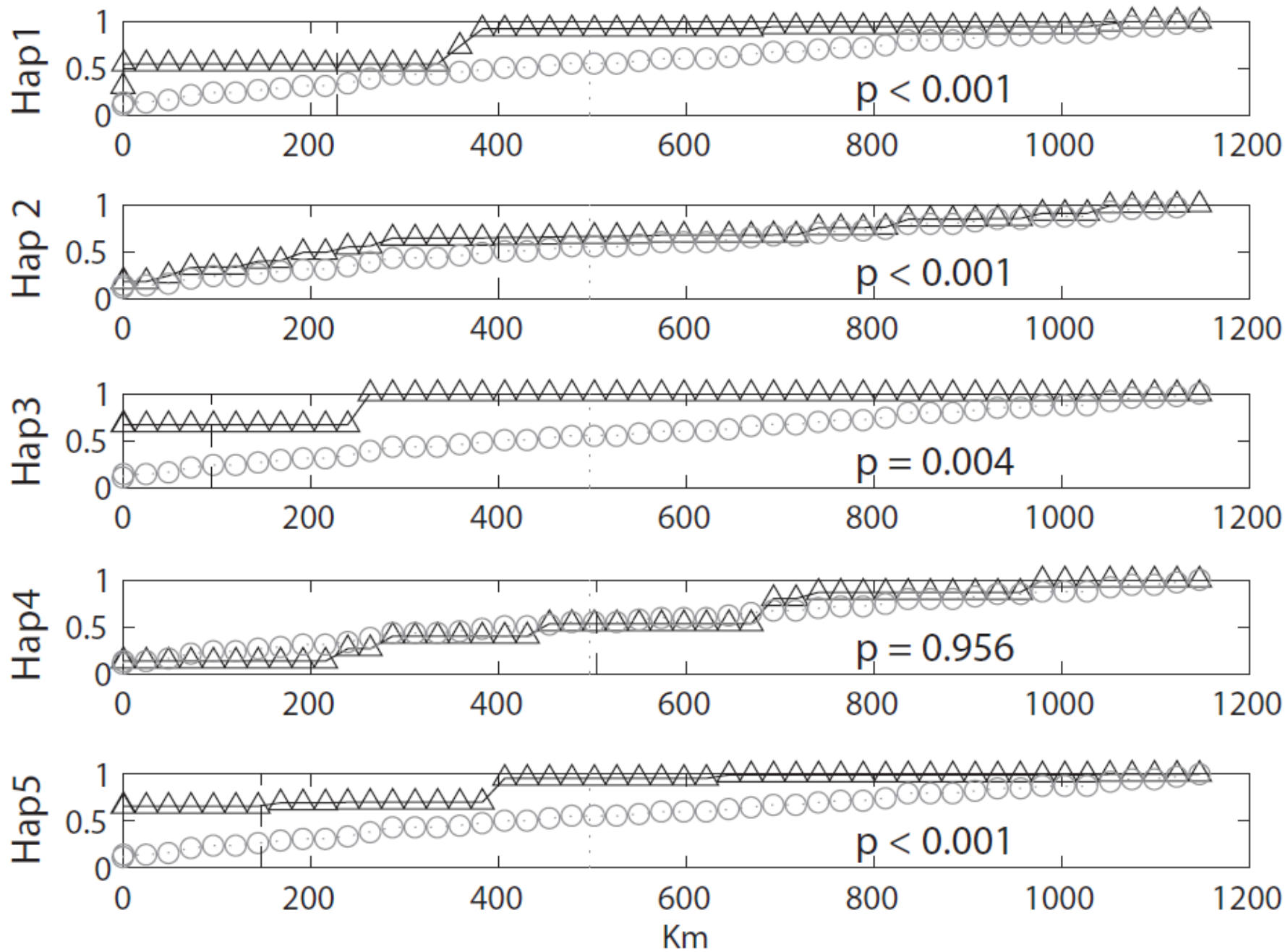

Supplement: Supplemental Information 2 — Frequency distributions of observed (triangles) and expected (circles) distances between co-occurrences of the alleles present in more than one locality, both sampling dates pooled. p-values indicate significant differences obtained through permutation of data. [file peerj-04-2158-s002.pdf]
