# Supplementary material for: Stochasticity in space, persistence in time: genetic heterogeneity in harbour populations of the introduced ascidian Styela plicata
Source: PeerJ. 2016 Jun 23;4:e2158. doi: 10.7717/peerj.2158 (PMC4924124; doi:10.7717/peerj.2158)
Supplement: Supplemental Information 3 [file peerj-04-2158-s003.docx]

|  | **Year** | **Hap_1** | **Hap_2** | **Hap_3** | **Hap_4** | **Hap_5** | **Hap_9** | **Hap_23** | **Hap_24** | **Hap_25** | **Hap_26** | **Hap_27** | **Hap_28** |
| --- | --- | --- | --- | --- | --- | --- | --- | --- | --- | --- | --- | --- | --- |
| **BLA** | **2009** | **18** | **1** |  |  |  | **1** | **1** |  |  |  |  |  |
|  | **2014** | **22** | **1** |  |  |  | **2** |  |  |  | **1** |  |  |
| **ARE** | **2009** | **19** | **1** |  |  |  |  |  |  |  |  |  |  |
|  | **2014** | **21** | **2** |  |  | **1** |  |  |  |  |  |  |  |
| **VIL** | **2009** |  | **21** |  |  |  |  |  | **1** |  |  |  |  |
|  | **2014** |  | **19** |  | **1** |  |  |  |  |  |  | **1** |  |
| **XAB** | **2009** | **14** |  | **4** | **2** |  |  |  |  |  |  |  |  |
|  | **2014** | **17** | **1** | **1** |  |  |  |  |  |  |  |  | **1** |
| **CAR** | **2009** |  | **2** |  |  | **18** |  |  |  | **1** |  |  |  |
|  | **2014** |  | **3** | **1** |  | **18** |  |  |  |  |  |  |  |
| **HER** | **2009** |  | **22** |  | **1** | **1** |  |  |  |  |  |  |  |
|  | **2014** |  | **21** |  |  |  |  |  |  |  |  |  |  |
| **TOR** | **2009** |  | **23** |  |  |  |  |  |  |  |  |  |  |
|  | **2014** |  | **21** |  |  |  |  |  |  |  |  |  |  |
| **CON** | **2009** | **2** | **19** |  | **1** | **2** |  |  |  |  |  |  |  |
|  | **2014** | **3** | **12** |  | **1** | **5** |  |  |  |  |  |  |  |
| **CHI** | **2009** |  | **24** |  |  |  |  |  |  |  |  |  |  |
|  | **2014** |  | **20** |  |  |  |  |  |  |  |  |  |  |
| **Total** |  | **116** | **213** | **6** | **6** | **45** | **3** | **1** | **1** | **1** | **1** | **1** | **1** |
